# Supplementary material for: Variability and uncertainty of data from genotoxicity test guidelines: what we know and why it matters
Source: Arch Toxicol. 2026 Feb 17;100(5):1713–34. doi: 10.1007/s00204-025-04277-9 (PMC13086873; doi:10.1007/s00204-025-04277-9)
Supplement: Supplementary file 1 — Supplementary file1 (PDF 278 KB) [file 204_2025_4277_MOESM1_ESM.pdf]

Supplement S1 to: **Variability and uncertainty of data from genotoxicity Test Guidelines: What we know and why it matters.**

Table S1a. OECD genotoxicity test guidelines (TGs) adaptations over time

OECD TG	Title	About	Adopted / Revised	New developments	Reference
**IN VITRO TGs**					
471	Bacterial Reverse Mutation Test	Evaluates the potential of chemicals to cause gene mutations in bacterial cells.	1983 / 1997 (and in 2020 only the CAS number of one of the positive controls as corrected)	• Increased documentation requirement and need for target tissue exposure and verification<br>• Negative and positive historical control data availability and use for data interpretation<br>• Increased documentation for lab proficiency but need for proficiency verification<br>• Addition of test species *E. coli*<br>• Better documentation of mutation types<br>• More explicit details on the species, strain, cultures tested<br>• Updated and more detailed exposure route regimen to cover different cases applicable<br>• More detailed exposure dose range; therefore also the data on sampling post application<br>• Increased documentation required for mean and standard deviation of reported parameters	OECD TG 471 (2020)
473	In vitro Mammalian Chromosomal Aberration Test	Evaluates the potential of chemicals to cause chromosomal damage in cultured mammalian cells *in vitro.*	1984 / 1997, 2014, 2016	• Increased documentation requirement and need for target tissue exposure and verification<br>• Negative and positive historical control data availability and use for data interpretation<br>• Increased documentation for lab proficiency<br>• More explicit requirements on the number of cell (not only scored, but treated, harvested, with CA) and its documentation Number and type of aberrations: need for more explicit requirement to report them<br>• Estimation of the mitotic index where applicable and more detailed<br>• Elaborate estimation of cytotoxicity parameters and indices<br>• Highest exposure dose estimation more detailed and including different cases<br>• Other effect values or observations have more detailed documentation and are required to be reported<br>• The exposure route regimen is updated and more detailed to cover different cases applicable<br>• Trend test explicitly mentioned for statistics	OECD TG 473 (2016)
476	In vitro Mammalian Cell Gene Mutation Test using the Hprt and xprt genes	The purpose of this test is to detect chemically-induced gene mutations in mammalian cells in	1984 / 1997, 2015, 2016	• The endpoint is more explicitly detailed based on the updated test objective<br>• Increased documentation requirement and need for target tissue exposure and verification	OECD TG 476 (2016)

		vitro (replaced by OECD TG 490).		• Negative and positive historical control data availability and use for data interpretation<br>• Increased documentation for lab proficiency<br>• Requirement of extensive estimation of cell numbers under different cultures or regimens<br>• Mutant frequency explicitly estimated<br>• Mutation types: need for more explicit requirement to report them<br>• The cell types and cell numbers in the culture/medium per dose are more detailed specified per case<br>• Elaborate estimation of cytotoxicity parameters and indices<br>• Highest exposure dose estimation more detailed and including different cases<br>• Other effect values or observations have more detailed documentation and are required to be reported<br>• The treatment schedules are updated and more detailed to cover different cases applicable<br>• The exposure duration more detailed explained<br>• The exposure dose range more detailed; also the estimation of highest exposure dose and different cases<br>• Increased documentation need for mean and standard deviation of reported parameters<br>• Trend test explicitly mentioned for statistics	
487	In vitro Mammalian Cell Micronucleus Test	This method assesses the potential of chemicals to induce micronuclei in cultured mammalian cells, a key indicator of chromosomal damage. When the test is combined with immunostaining labelling of kinetochores or hybridisation with centromeric/telomeric probes (FISH) can provide additional information on the mechanism of chromosome damage.	2010 / 2014, 2023	• Increased documentation requirement and need for target tissue exposure and verification<br>• Negative and positive historical control data availability and use for data interpretation<br>• Increased documentation for lab proficiency<br>• More detailed documentation of micronuclei/aberration: test for metaphase chromosome aberrations<br>• Highest exposure dose estimation more detailed and including different cases<br>• Cell lines applicable in the test and cell numbers in cultures are more detailed documented<br>• Increased documentation need for mean and standard deviation of reported parameters<br>• Trend test explicitly mentioned for statistics	OECD TG 487 (2023)
490	In vitro Mammalian Cell Gene Mutation Test Using the	The purpose of this test is to detect chemically-induced gene mutations in	2015 / 2016	Not applicable. The test guideline describes separately criteria and conditions for MLA and TK6.	OECD TG 490 (2016)

	Thymidine Kinase Gene	mammalian cells (mouse lymphoma cell line and TK6 human lymphoblastoid cell line) in vitro (replaces OECD TG 476).			
**IN VIVO TGs**					
470	Mammalian Erythrocyte Pig-a Gene Mutation Assay	This is an in vivo genotoxicity test that measures mutations in the Pig-a gene in peripheral blood reticulocytes or erythrocytes of rodents to assess the potential for chemical-induced gene mutations.	2022	Not applicable; not revised	OECD TG 470 (2022)
474	Mammalian Erythrocyte Micronucleus Test	A standard test used to assess the potential of chemicals to induce chromosomal damage by detecting micronucleus formation in bone marrow cells of mammals in vivo.	1983 / 1997, 2014, 2016	• Automated scoring<br>• Increased documentation need for target tissue exposure<br>• Negative and positive historical control data availability and use for data interpretation<br>• Increased documentation for lab proficiency<br>• Increased number of PCE screened for MN detection<br>• Increased number of cells for PCE/NCE ratio determination and use of PCE count relative to control to determine toxicity (min 20% of control)<br>• Analysis for clastogenicity or aneuploidy (kinetochor stain with antibody or DNA probe)<br>• Trend test explicitly mentioned for statistics	OECD TG 474 (2016)
475	Mammalian Bone Marrow Chromosomal Aberration Test	This test detects chromosomal aberrations induced by chemicals in the bone marrow of mammals in vivo.	1984 / 1997, 2014, 2016	• Increased documentation requirement and need for target tissue exposure<br>• Negative and positive historical control data availability and use for data interpretation<br>• Increased documentation for lab proficiency<br>• Number of metaphases scored (not only cell number)<br>• Number and type of aberrations: need for more explicit requirement to report them<br>• Estimation of the mitotic index where applicable<br>• Cell and/or centromere scoring and documentation is more explicitly required<br>• The species, strain, sex, and numbers of animals tested are more explicitly detailed.	OECD TG 475 (2016)

				• The exposure route regimen is updated and more detailed to cover different cases applicable<br>• The exposure dose range more detailed; therefore also the data on sampling post application<br>• increased documentation need for mean and standard deviation of reported parameters<br>• Verification of exposure of the target tissue<br>• MTD as max dose explicitly required in the dose levels<br>• Trend test explicitly mentioned for statistics	
478	Rodent Dominant Lethal test	The purpose of this test is to investigate whether chemicals produce mutations resulting from chromosomal aberrations in germ cells.	1984 / 2015, 2016	• Increased documentation requirement and need for target tissue exposure<br>• Negative and positive historical control data availability and use for data interpretation<br>• Increased documentation for lab proficiency<br>• Number of implants, embryos and other parameters scored (not only cell number)<br>• Number of parameters linked with the pre and post implantation loss (e.g. corpora lutea per dam)<br>• More detailed documentaiton of data linked with DL frequencies<br>• The strain, and numbers of animals tested are more explicitly detailed according to statistical power.<br>• Animal body weight and food consumption recording: need for documentation<br>• The exposure route regimen is updated and more detailed to cover different cases applicable<br>• The exposure dose range more detailed; therefore also the data on sampling post application<br>• The exposure duration and regimens are updated and more detailed<br>• Increased documentation need for mean and standard deviation of reported parameters<br>• Verification of exposure of the target tissue<br>• MTD as max dose explicitly required in the dose levels<br>• Trend test explicitly mentioned for statistics	OECD TG 487 (2016)
483	Mammalian Spermatogonial Chromosome Aberration Test	This test detects chemicals that induce structural chromosome- and chromatid-type aberrations in dividing	1986 / 1997, 2015	• Increased documentation requirement and need in reporting and confirmation for target tissue exposure<br>• Negative and positive historical control data availability and use for data interpretation	OECD TG 483 (2016)

		mammalian spermatogonial germ cells in vivo.		• Increased documentation for lab proficiency<br>• Number of metaphases scored (not only cell number)<br>• Number and type of aberrations: need for more explicit requirement to report them<br>• Estimation of the mitotic index where applicable<br>• Increased documentation need for mean and standard deviation of reported parameters<br>• Increased documentation need for organ weight data/measurements<br>• Cell and/or centromere scoring and documentation is more explicitly required<br>• The species, weight variation, and numbers of animals tested are more explicitly detailed.<br>• The exposure route regimen is updated and more detailed to cover different cases applicable.<br>• Verification of exposure of the target tissue<br>• MTD as max dose explicitly required in the dose levels	
485	Genetic toxicology, Mouse Heritable Translocation Assay	This test aims at detecting structural and numerical chromosome changes in mammalian germ cells as recovered in first generation progeny (reciprocal translocations and, if female progeny are included, X-chromosome loss)	1986	Not applicable; not revised	OECD TG 485 (1986)
488	Transgenic Rodent Somatic and Germ Cell Gene Mutation Assay	This test is designed to detect gene mutations in the DNA of transgenic rodents and is used to evaluate the mutagenic potential of chemicals in vivo.	2011 / 2013, 2022	• Increased documentation requirement in reporting for target tissue exposure<br>• Negative and positive historical control data availability and use for data interpretation<br>• Increased documentation for lab proficiency<br>• The age range of animals at the start of the treatment (and regimen regarding sex of animals)<br>• The reproductive tracts to be sampled for sperm collection updated<br>• The time for rodent spermatogonial stem cells to become mature sperm and reach the cauda epididymis<br>• The recommended regimen for the analysis of mutations in male sperm gells is extensively detailed affecting sampling schemes post applications<br>• Verification of exposure of the target tissue	OECD TG 488 (2022)

				• MTD as max dose explicitly required in the dose levels<br>• Trend test explicitly mentioned for statistics	
489	In vivo Mammalian Alkaline Comet Assay	The test is used for the detection of DNA strand breaks in cells or nuclei isolated from multiple tissues of animals (usually rodents) after exposure to chemicals in vivo.	2014 / 2016	The overview of the set of OECD Genetic Toxicology Test Guidelines and updates performed in 2014-2015 (OECD, 2016) has been considered, but the text in the TG was not revised	OECD TG 489 (2016)
**Archived and deleted OECD TGs**					
**IN VIVO TGs**					
472	Genetic toxicology: Escherichia coli, Reverse Assay		1983 / deleted 1997	Not applicable	OECD TG 472 (1983)
477	Sex-linked recessive lethal test in Drosophila melanogaster		1984 / deleted 2014	Not applicable	OECD TG 477 (1984)
479	In vitro sister chromatid exchange assay in mammalian cells		1986 / deleted 2014	Not applicable	OECD TG 479 (1986)
480	Saccharomyces cerevisiae, gene mutation assay		1986 / deleted 2014	Not applicable	OECD TG 480 (1986)
481	Saccharomyces cerevisiae, mitotic recombination assay		1986 / deleted 2014	Not applicable	OECD TG 481 (1986)
482	DNA damage and repair, unscheduled DNA synthesis in mammalian cells in vitro		1986 / deleted 2014	Not applicable	OECD TG 482 (1986)
**IN VIVO TGs**					
484	Mouse spot test		1986 / deleted 2014	Not applicable	OECD TG 484 (1986)
486	Unscheduled DNA synthesis (UDS) test with mammalian liver cells in vivo		1997 /deleted 2020	Not applicable	OECD TG 486 (1997)

Table S1b. Adaptations within the different OECD genotoxicity TGs over time

**Improvement**	**Subcategory**	**Type of study**	**OECD TG**
**Improved documentation and reporting standards**	**Improved documentation of species, strains, cultures**	**In vitro**	**OECD TG 471:** More explicit detailing of species, strain, and cultures tested<br>**OECD TG 476**: More detailed specification of cell types and cell numbers in cultures per dose
		**In vivo**	**OECD TG 488**: Documentation of the age range of animals at the start of treatment and sex regimen<br>**OECD TG 478**: More explicit detailing of strain and number of animals based on statistical power
	**Improved documentation of exposure**	**In vitro**	**OECD TG 471**: More detailed exposure dose range and post-application sampling data<br>**OECD TG 471, 473**: Updated exposure route regimen<br>**OECD TG 476**: Detailed treatment schedules, exposure duration, and dose range estimations<br>**OECD TG 473, 476, 487**: More detailed estimations of the highest exposure dose
		**In vivo**	**OECD TG 475, 478**: Updated and more detailed exposure route regimen<br>**OECD TG 478, 483, 488**: MTD explicitly required in dose levels<br>**OECD TG 475, 478, 483**: More detailed exposure dose range, sampling post-application, and target tissue exposure verification
	**Enhanced cell scoring and documentation**	**In vitro:**	**OECD TG 473**: Detailed reporting on the number of cells (scored, treated, harvested) and chromosomal aberrations<br>**OECD TG 476:** Extensive estimation of cell numbers under different conditions
		**In vivo:**	**OECD TGs 483, 475**: Documentation includes number of metaphases scored<br>**OECD TG 478**: Includes the number of implants, embryos, and other parameters scored

			**OECD TG 474**: Automated scoring, increased PCE screening for MN detection, and increased cells for PCE/NCE ratio determination<br>**OECD TG 488**: Updates on reproductive tract sampling, sperm collection, and timing for spermatogonial stem cells maturation
	**Improved documentation of results**	**In vitro**	**OECD TG 471**: Detailed documentation of mutation types<br>**OECD TG 473**: Number and types of aberrations, mitotic index estimation, and cytotoxicity parameters.<br>**OECD TG 487**: More detailed documentation of micronuclei/aberration tests and cell line details
		**In vivo**	**OECD TG 483, 475**: Cell/centromere scoring and documentation, number and types of aberrations, mitotic index estimation<br>**OECD TG 474**: Analysis for clastogenicity/aneuploidy<br>**OECD TG 475**: More explicit reporting of species, strain, sex, and number of animals tested, as well as MTD in dose levels<br>**OECD TG 478**: Documentation of dose-level frequencies and pre/post-implantation loss parameters
**Advances in testing methodologies**	**Improved experimental protocol**	**In vitro**	**OECD TG 471**: Test species E. coli added.<br>**OECD TG 473**: Mitotic index and cytotoxicity estimation, highest esposure dose estimation, detailed scoring of aberrations<br>**OECD TG 476:** Updated protocols for exposure dose selection and range, treatment schedule and duration, cytotoxicity estimation
		**In vivo**	**OECD TG 478**: Documentation of animal body weight and food consumption<br>**OECD TG 483**: Increased documentation for organ weight data and animal details (species, weight variation, and numbers
	**Improved statistical and analytical methods**	**In vitro**	**OECD TGs 471, 473, 476, 487**
		**In vivo**	**OECD TGs 474, 475, 478, 483**
	**Incorporation of historical control data**	**In vitro**	**OECD TGs 471, 473, 476, 487**
		**In vivo**	**OECD TGs 474, 475, 478, 483, 488**
	**Focus on laboratory proficiency and quality assurance**	**In vitro**	**OECD TGs 471, 473, 476, 487**
		**In vivo:**	**OECD TGs 474, 475, 478, 483, 488**
	**Adaptation of new scientific insights**	**In vitro**	**OECD TGs 473, 487: c**lastogenicity, aneuploidy, cytotoxicity
		**In vivo**	**OECD TG 488:** timing for spermatogonial stem cells maturation in rodents

Table S1c: Overview on OECD documents screened for information on references possibly informing on data variability.

OECD Documents	Number of references in the document	References possibly informing on data variability
TG 470	65	15
TG 471	24	5
TG 476	48	4
TG 488	61	7
TG 490	67	18
T&A 103	ca 600	n.a.
T&A 145	23	13
T&A 224	0	0
T&A 315	Ca 300	n.a.
T&A 316	38	8
T&A 319	0	0
T&A 358	95	11
TG 486	6	3
TG 489	73	18
T&A 195	19	5
T&A196	ca 150-200	n.a.

T&A 197	20	5
TG 473	55	10
TG 474	47	14
TG 475	13	5
TG 483	22	3
TG 487	93	8
T&A 198	15	4
GD 238	Ca 130-160 n.a.	1

n.a.= not analysed here; T&A: OECD Series of Testing and Assessment
