# Supplementary material for: Variability and uncertainty of data from genotoxicity test guidelines: what we know and why it matters
Source: Arch Toxicol. 2026 Feb 17;100(5):1713–34. doi: 10.1007/s00204-025-04277-9 (PMC13086873; doi:10.1007/s00204-025-04277-9)
Supplement: Supplementary file 3 — Supplementary file3 (PDF 653 KB) [file 204_2025_4277_MOESM3_ESM.pdf]

Supplement S2b to **Variability and uncertainty of data from genotoxicity Test Guidelines: What we know and why it matters.**

Application of the RF technique to the other three subsets of data, i.e. the Mammalian Bone Marrow Chromosome Aberration Test (OECD Guideline 475), the Mammalian Chromosome Aberration Test - OECD Guideline 473 and in vitro Mammalian Cell Gene Mutation Test - OECD Guideline 476.

1 Mammalian Bone Marrow Chromosome Aberration Test (OECD Guideline 475)

In the case of the Mammalian Bone Marrow Chromosome Aberration Test (OECD Guideline 475), the dataset comprises 348 entries for 67 individual compounds and the following variables were considered:

- SPECIES (Hamster (as animal), Rat (as animal), House mouse (as animal) and Rabbit (as animal);
- STRAIN (Chinese, Syrian or not reported in the case of Hamster; CD-1, B6C3F1, Swiss, not reported, C57BL, ICR, SwissWebster and CF-1 in the case of House mouse; not specified in the case of Rabbit; Fischer344, Sprague-Dawley, Crj:CD(SD), Wistar, Alderly Park Rat in the case of Rat);
- NUMBER_INDIVIDUALS (from 2 to 92);
- SEX (Male/Female, Male and Female);
- ROUTE (oral: gavage, oral: unspecified, inhalation: unspecified, intraperitoneal, oral: feed, subcutaneous and oral: drinking water);
- EXP_PERIOD (from 2 hours to 14 weeks);
- RESULTS (negative, positive, ambiguous and inconclusive);
- opinion_pub_year;
- literature_reference years

Of the 67 individual compounds, 14 have data with multiple in vivo Chromosome Aberration results (equivocal, inconclusive, negative and positive outcomes)

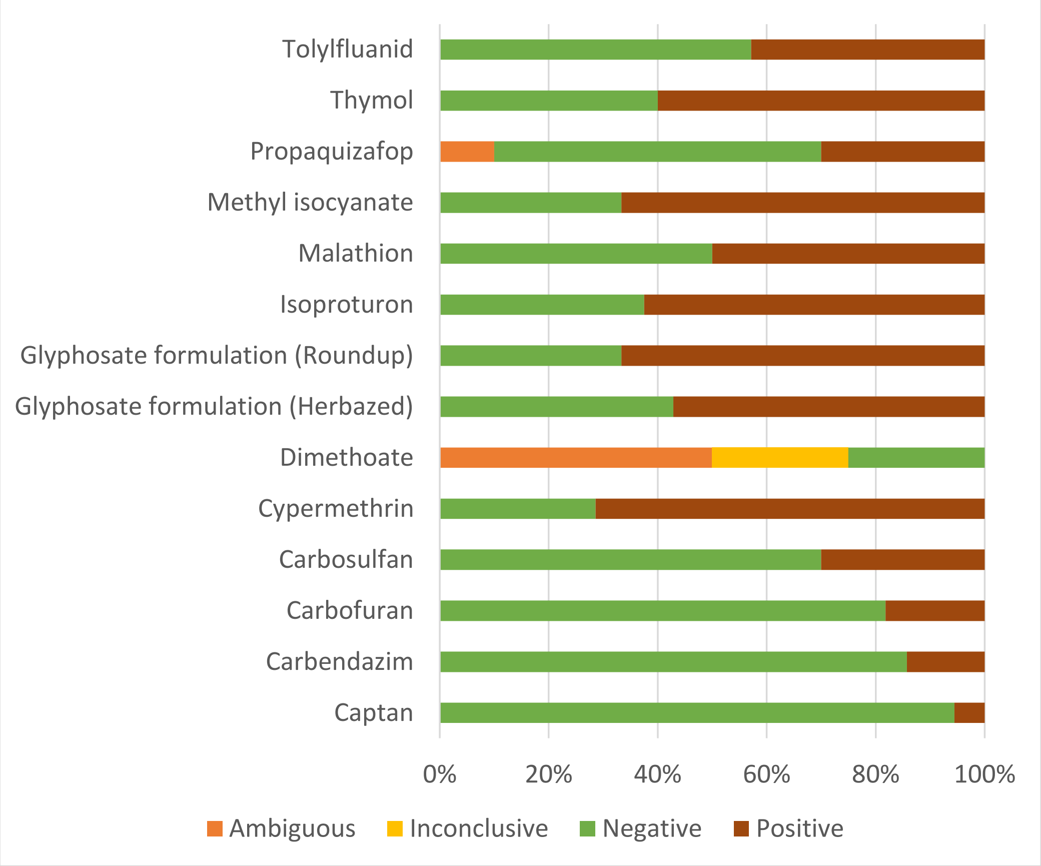

Fig. S3.1. Distribution of the 14 compounds with multiple in vivo chromosome aberration results.

Supplement S2b to **Variability and uncertainty of data from genotoxicity Test Guidelines: What we know and why it matters.**

The RF technique identified the most relevant variables associated with the different results for 10 of these 14 compounds. More than one variable is involved in the different outcomes for the same compound. However, for Glyphosate formulations (Roundup), Isoproturon, Methyl isocyanate and Propaquizafop, none of the variables were selected to explain the different results.

Table S3.1, Distribution of the relevant variables for the 10 compounds

**Name of the compound**	**variable n. 1**	**variable n. 2**	**variable n. 3**
Captan	strain	literature_reference	
Carbendazim	species	strain	literature_reference
Carbofuran	number_individuals		
Carbosulfan	number_individuals		
Cypermethrin	species	strain	
Dimethoate	strain	sex	
Glyphosate formulation (Herbazed)	route		
Malathion	species	number_individuals	route
Thymol	number_individuals	route	literature_reference
Tolylfluanid	number_individuals	literature_reference	

Considering the number of compounds, the most relevant variables are NUMBER_INDIVIDUALS (5/10, 50% of coverage), STRAIN and literature_reference (4/10, 40 % of coverage)

Supplement S2b to **Variability and uncertainty of data from genotoxicity Test Guidelines: What we know and why it matters.**

2 In vitro Mammalian Chromosome Aberration Test - OECD Guideline 473

The variables considered as input to the RF technique for the in vitro data sets (Mammalian Chromosome Aberration Test - OECD Guideline 473 and in vitro Mammalian Cell Gene Mutation Test - OECD Guideline 476) were obviously slightly different from those in vivo. The following are missing: NUMBER_INDIVIDUALS, SEX, ROUTE, EXP_PERIOD and INVIVOTISSUEEXP. A new variable has been added: MET_INDICATOR.

Table S3.2. Variables considered as input of the RF technique for in vitro Mammalian Chromosome Aberration Test - OECD Guideline 473 dataset.

SPECIES	STRAIN	MET_INDICATOR	RESULTS	opinion_pub_year	literature_reference
Hamster (as animal)	CHO	with	Negative	Years from 2005 to 2016	217 documents
Human (as organism)	lung fibroblasts (V79)	without	Positive		
Rat (as animal)	lymphocytes		Ambiguous		
House mouse (as animal)	spleen cells		Inconclusive		
Bovinae (bovines = Tribe bovini) (as animal)	lymphoma L5178Y cells				
	fibroblast				
	embryo cells				
	hepatocytes				

This first in vitro dataset comprises 452 entries for 97 individual compounds. Of these, 49 had multiple results from the Mammalian Chromosome Aberration Test (including equivocal, inconclusive, negative and positive outcomes).

Supplement S2b to **Variability and uncertainty of data from genotoxicity Test Guidelines: What we know and why it matters.**

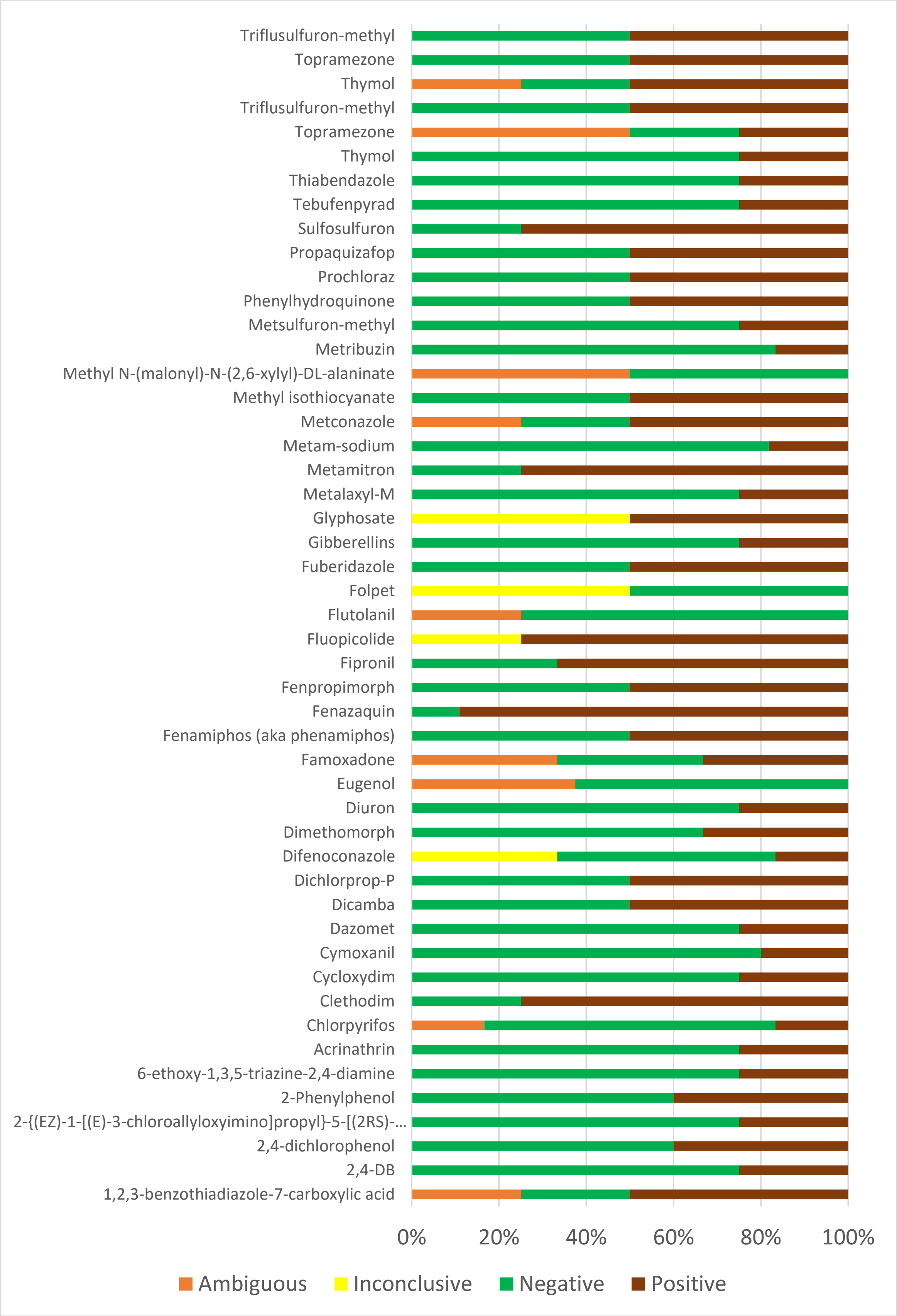

Fig. S3.2. Distribution of the 49 compounds with multiple in vivo chromosome aberration results.

Supplement S2b to **Variability and uncertainty of data from genotoxicity Test Guidelines: What we know and why it matters.**

For 18 compounds of these 49, the RF technique identified the most relevant variables related to the different outcomes.

Table S3.3. Distribution of the relevant variables for the 18 compounds.

**Name of the compound**	**variable n. 1**	**variable n. 2**
2-Phenylphenol	strain	
2-{(EZ)-1-[(E)-3-chloroallyloxyimino]propyl}-5-[(2RS)-2-(ethylsulfonyl)propyl]-3-hydroxycyclohex-2-en-1-one	met_indicator	
Acrinathrin	strain	
Bentazone	literature_reference	
Bromuconazole	met_indicator	literature_reference
Chlorpyrifos	strain	literature_reference
Clethodim	met_indicator	
Difenoconazole	species	
Dimethomorph	species	strain
Fenazaquin	literature_reference	
Fuberidazole	met_indicator	
Gibberellins	literature_reference	
Metconazole	species	
Methyl N-(malonyl)-N-(2,6-xylyl)-DL-alaninate	met_indicator	
Methyl isothiocyanate	species	strain
Prochloraz	met_indicator	
Propaquizafop	met_indicator	
Sulfosulfuron	strain	

Considering the number of compounds, MET_INDICATOR has the highest coverage (7/18, 39%) followed by STRAIN (6/18, 33% of coverage) andliterature_reference (5/18, 28% of coverage). For the following 31 compounds none of the variables were selected to explain the different results:

1,2,3-benzothiadiazole-7-carboxylic acid 2,4-DB 2,4-dichlorophenol, 6-ethoxy-1,3,5-triazine-2,4-diamine, Carfentrazone-ethyl, Cycloxydim, Cymoxanil, Dazomet, Dicamba, Dichlorprop-P, Diuron, Eugenol, Famoxadone, Fenamiphos (aka phenamiphos), Fenpropimorph, Fipronil, Fluopicolide, Flutolanil, Folpet, Glyphosate, Metalaxyl-M, Metamitron, Metam-sodium, Metribuzin, Metsulfuron-methyl, Phenylhydroquinone, Tebufenpyrad, Thiabendazole, Thymol, Topramezone, Triflusulfuron-methyl.

3 In vitro Mammalian Cell Gene Mutation Test (OECD Guideline 476)

The dataset of in vitro Mammalian Cell Gene Mutation Test (OECD Guideline 476) counts 1365 entries for 482 single compounds. Out of these 482 individual compounds, 61 had multiple data (equivocal, inconclusive, negative and positive results). The variables considered as input of the RF technique were:

- SPECIES (House mouse (as animal), Hamster (as animal), Human (as organism);
- STRAIN (lymphoma L5178Y cells CHO, lung fibroblasts (V79), embryo cells, lymphocytes);
- MET_INDICATOR (with and without);
- mouselymphtest (small colonies and large colonies);
- RESULTS (negative, positive, ambiguous and inconclusive);
- opinion_pub_year (2005-2016);
- literature_reference years (600 documents).

Supplement S2b to **Variability and uncertainty of data from genotoxicity Test Guidelines: What we know and why it matters.**

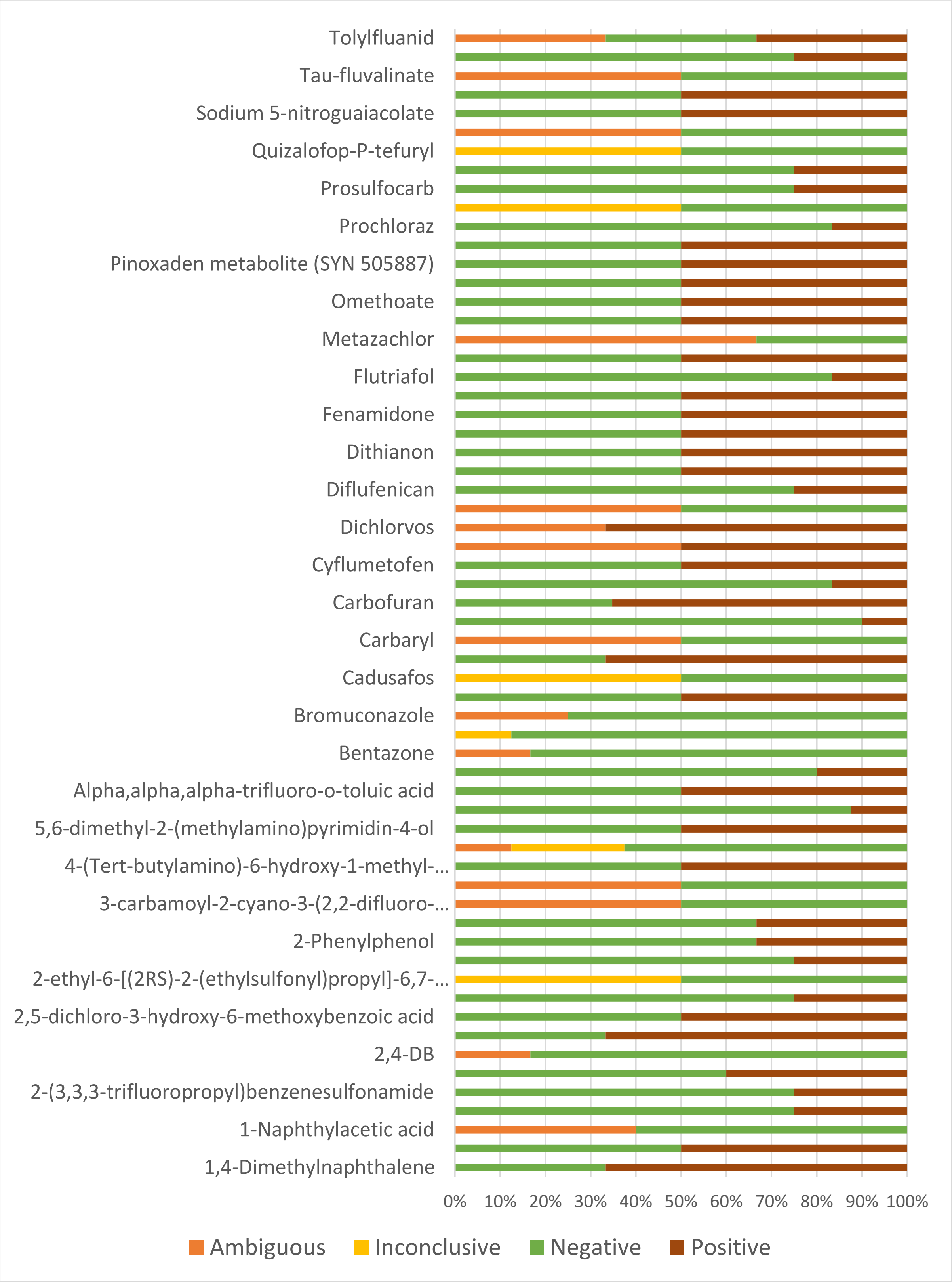

Fig. S3.3. Distribution of the 61 compounds with multiple in vitro Mammalian Cell Gene Mutation Test results.

Supplement S2b to **Variability and uncertainty of data from genotoxicity Test Guidelines: What we know and why it matters.**

For 10 compounds of these 61, the RF technique identified the most relevant variables related to the different outcomes. Also in this case*,* more than one variable occurs (are influent/relevant in the case of different results) for the same compound.

Table S3.4. 10 compounds with multiple results explained by the RF

**Name of compound**	**variable n. 1**	**variable n. 2**	**variable n. 3**
2-(3,3,3-trifluoropropyl)benzenesulfonamide	met_indicator		
4-methoxy-6-methyl-1,3,5-triazine-2-amine	met_indicator		
Bentazone	met_indicator		
Bifenthrin	species	strain	literature_reference
Carbofuran	strain		
Cycloxydim	species		
Flutriafol	literature_reference		
Metazachlor	species	strain	literature_reference
Terbuthylazine	strain		
Tolylfluanid	species		

Considering the number of compounds, the variables have similar coverage:

"met_indicator" and "literature_reference" have 30% of coverage (3/10), meanwhile "species" and "strain" have higher coverage 40% (4/10). For the following 31 compounds none of the variables were selected to explain the different results:

1,4-Dimethylnaphthalene, 1-Methyl-3-trifluoromethyl-1H-pyrazole-4-carboxamide, 1-Naphthylacetic acid, 2-(1-Naphthyl)acetamide, 2,4-D, 2,4-DB, 2,4-dichlorophenol, 2,5-dichloro-3-hydroxy-6-methoxybenzoic acid, 2-{(EZ)-1-[(E)-3-chloroallyloxyimino]propyl}-5-[(2RS)-2-(ethylsulfonyl)propyl]-3-hydroxycyclohex-2-en-1-one, 2-ethyl-6-[(2RS)-2-(ethylsulfonyl)propyl]-6,7-dihydro-1,3-benzoxazol-4(5H)-one, 2-hydroxynaphthalene-1,4-dione, 2-Phenylphenol, 3-(trifluoromethyl)benzoic acid, 3-carbamoyl-2-cyano-3-(2,2-difluoro-benzo[1,3]dioxol-4-yl)-oxirane-2-carbocyclic acid, 3-chloro-1-methyl-5-sulfamoyl-1H-pyrazole-4-carboxylic acid, 4-(Tert-butylamino)-6-hydroxy-1-methyl-1,3,5-triazin-2(1H)-one, 5,6-dimethyl-2-(methylamino)pyrimidin-4-ol, 6-Benzyladenine, Alpha,alpha,alpha-trifluoro-o-toluic acid, Amitrole, Bromuconazole, Butyrac 200, Cadusafos, Captan, Carbaryl, Carbendazim, Cyflumetofen, Dazomet, Dichlorvos, Dicloran, Diflufenican, Diphenylamine, Dithianon, Ethoprophos, Fenamidone, Fluroxypyr-meptyl, Hymexazol, Napropamide, Omethoate, Phosmet, Pinoxaden metabolite (SYN 505887), Pirimicarb, Prochloraz, Proquinazid, Prosulfocarb, Pyraflufen-ethyl, Quizalofop-P-tefuryl, Racemic Haloxyfop, Sodium 5-nitroguaiacolate, Sulcotrione, Tau-fluvalinate.
